# Supplementary material for: Time‑resolved multi-omic analysis of paclitaxel exposure in human iPSC‑derived sensory neurons unveils mechanisms of chemotherapy‑induced peripheral neuropathy
Source: Cell Death Dis. 2026 Feb 10;17(1):211. doi: 10.1038/s41419-026-08445-2 (PMC12921266; doi:10.1038/s41419-026-08445-2)
Supplement: Supplementary file 15 — Dataset 1. [file 41419_2026_8445_MOESM15_ESM.zip › CDD_Open_Data/04_RNA_Seq/Timeline_panel_plots_etc_2023-01-23_1609.zip!/Panel_Plots_etc/Evidence_Plots/6h/evidence_plot_standard contrasts_msigdb_reactome_6h_PTX_vs_DMSO_ID2_M11716_REACTOME_FORMATION_OF_ATP_BY_CHEMIOSMOTIC_COUPLING.pdf]

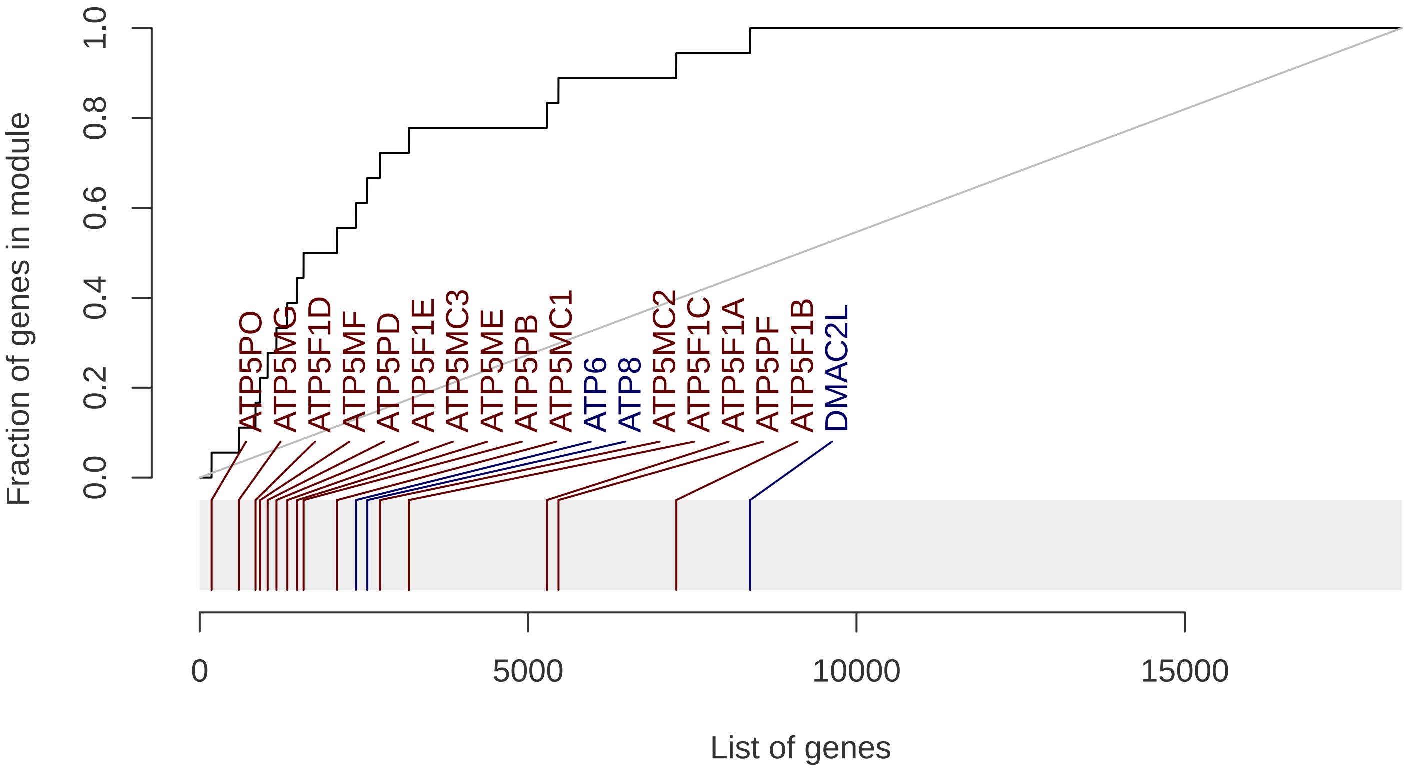
Fraction of genes in module
0.0
0.2
0.4
0.6
0.8
1.0
ATP5PO
ATP5MG
ATP5F1D
ATP5MF
ATP5PD
ATP5F1E
ATP5MC3
ATP5ME
ATP5PB
ATP5MC1
ATP6
ATP8
ATP5MC2
ATP5F1C
ATP5F1A
ATP5PF
ATP5F1B
DMAC2L
0
5000
10000
15000
List of genes
